# Elephant movement closely tracks precipitation-driven vegetation dynamics in a Kenyan forest-savanna landscape

**DOI:** 10.1186/2051-3933-2-2

**Published:** 2014-01-29

**Authors:** Gil Bohrer, Pieter SA Beck, Shadrack M Ngene, Andrew K Skidmore, Ian Douglas-Hamilton

**Affiliations:** The Ohio State University, Department of Civil, Environmental and Geodetic Engineering, 2070 Neil Ave., Columbus, OH 43210 USA; Woods Hole Research Center, 149 Woods Hole Road, Falmouth, MA 02540-1644 USA; Kenya Wildlife Service, Meru National Park, P.O. Box 11, Maua, Kenya; International Institute of Geo-information Science and Earth Observation, Hengelosestraat 99, P.O. Box 6, 7500 AA Enschede, The Netherlands; Save the Elephant Trust, P.O. Box 54667, Nairobi, 00200 Kenya

**Keywords:** Seasonality, Rainfall, Human-elephant conflict, Remote sensing, NDVI

## Abstract

**Background:**

This study investigates the ranging behavior of elephants in relation to precipitation-driven dynamics of vegetation. Movement data were acquired for five bachelors and five female family herds during three years in the Marsabit protected area in Kenya and changes in vegetation were mapped using MODIS normalized difference vegetation index time series (NDVI). In the study area, elevations of 650 to 1100 m.a.s.l experience two growth periods per year, while above 1100 m.a.s.l. growth periods last a year or longer.

**Results:**

We find that elephants respond quickly to changes in forage and water availability, making migrations in response to both large and small rainfall events. The elevational migration of individual elephants closely matched the patterns of greening and senescing of vegetation in their home range. Elephants occupied lower elevations when vegetation activity was high, whereas they retreated to the evergreen forest at higher elevations while vegetation senesced. Elephant home ranges decreased in size, and overlapped less with increasing elevation.

**Conclusions:**

A recent hypothesis that ungulate migrations in savannas result from countervailing seasonally driven rainfall and fertility gradients is demonstrated, and extended to shorter-distance migrations. In other words, the trade-off between the poor forage quality and accessibility in the forest with its year-round water sources on the one hand and the higher quality forage in the low-elevation scrubland with its seasonal availability of water on the other hand, drives the relatively short migrations (the two main corridors are 20 and 90 km) of the elephants. In addition, increased intra-specific competition appears to influence the animals’ habitat use during the dry season indicating that the human encroachment on the forest is affecting the elephant population.

## Background

For the successful conservation and management of migrating wildlife populations it is crucial to understand when animals move, where they move and why they move [1–3]. Failure to understand migration dynamics and their drivers jeopardizes the successful protection of animals and is likely to increase animal-human conflicts [4, 5]. Elephants play an important role in East-African ecosystems, both ecologically and as a source of revenue via tourism [6]. Their activity can dramatically affect vegetation composition and structure, in particular of woody species [7–10]. Consequently, it also modifies animal biodiversity [11, 12], as well as nutrient cycling and ecosystem productivity [13]. Moreover, elephants are responsible for crop-raiding, especially where cultivated land borders protected areas [14–16]. Hence, an improved understanding of the migrations of elephants, and how they relate to variation in their environment in space and time, will assist conservation and management of elephants and their habitats, as well as the management of adjacent farms.

The study of elephants’ migration has considered home range size [17–21], elephants’ travelling speed [22, 23] and has described differences and movement between elephants’ seasonal habitats [24, 25]. Murwira and Skidmore [26] showed that vegetation heterogeneity and patch size, estimated using remote sensing, are good predictors of elephant presence in savannah landscapes in northwestern Zimbabwe. Savanna elephants in Northern Kenya as well as desert-dwelling elephants in Namibia range over larger areas during wet seasons, when water sources are more prevalent, than during dry seasons [27, 28]. In addition, the latter population changed their foraging areas when artificial water points were built in their otherwise very dry habitat (< 100 mm annual rainfall, [29]). This influence of water holes on seasonal elephant ranging has also been documented in other arid areas along with effects of the erection of fences [30]. Clearly, the availability of forage and water, both natural and artificial, combined with other anthropogenic alterations of the landscape are key drivers in elephants’ habitat utilization and linking them to elephant ecology is essential to conservation.

In the past, data availability has forced a trade-off between describing the link between animal movement and landscape dynamics using either fine temporal or fine spatial resolutions. Equipping animals with GPS receivers provides animal movement data at daily or hourly temporal resolution and a spatial accuracy of meters [31]. These data can now be used to relate animal movements to changes of land cover and weather, owing to the availability of coincident satellite imagery that depict landscape patterns through time (e.g., [30, 32–34]).

Here, we analyze how elephant movement and habitat use relate to vegetation dynamics derived from the Normalized Difference Vegetation Index (NDVI) in a forest-savanna landscape in Kenya. Our study employs a framework for data visualization and analysis that links the movement of animals to changes in vegetation productivity through the landscape as well as through time [35]. In particular, we focus on the elephant population in the Marsabit protected area, which contains both forested and scrubland areas intermixed with settlements and farm land. While the lack of a detailed vegetation map for this area, prevented us from assessing the role of vegetation structure plays in animal movement, the NDVI serves as an index of vegetation conditions within the elephant range. The human population in the Marsabit has grown from 17,000 in 1979 to 43,000 in 2006, with an even more striking expansion of cropland, from 3596 ha in 1973 to 30,000 ha in 2005 [36]. While historic data for the Marsabit area are rare, this expansion has in all likelihood reduced the ranging areas of elephants and increased farmer-elephant conflicts. This is supported by reports of illegal killing of elephants in the area, while recent research indicates that elephants in the Marsabit area move faster in periods when livestock are herded in their habitat [37]. This evidence indicates that by understanding the movement patterns of elephants, the successful co-existence of humans and elephants may be promoted.

Satellite remote sensing data are ideally suited for spatio-temporal change analysis of landscapes. For green vegetation in particular, optical data from satellites has proven its capability to estimate the amount of green biomass in the landscape [38]. The NDVI exploits a contrast in reflectance in the near-infrared (Rnir), and red portions of the electromagnetic spectrum (Rred) which is typical of photosynthetically active vegetation. Hence, NDVI, defined as (Rnir-Rred)/(Rnir + Rred), is positively correlated with photosynthetically active biomass. Although NDVI values saturate in high biomass conditions [39] and are insensitive to changes in understory vegetation under a closed canopy [40], time series of NDVI reflect seasonal greening and senescing of vegetation in low to intermediate biomass conditions [41–44].

Relationships between elephant movement and vegetation density, as can be approximated by NDVI, were demonstrated before (e.g., [30, 45, 46]). These studies found large variation between individuals and other factors such as fences and human interactions to play a large role and therefore reduce the predictive power of NDVI and a driver for elephant location and movement patterns. In this study, we focus on determining the predictive power of NDVI on elephant movement. NDVI is a convenient driver for any movement model and particularly for use by wildlife managers of large areas because it relates well to the physical environment and it is available at high spatial and decent temporal resolution from remote sensing. Other drivers, such as social status, vegetation type or forage quality will demand large ground-based campaigns in order to obtain their values at a sufficient level of detail.

We combined time series of NDVI data with ground based meteorological data and the GPS-measured movement of ten elephants between 2005 and 2008. We first assess how well NDVI time-series capture the local and short-term rainfall-vegetation dynamics in the Marsabit area and how well they reflect the spatial and temporal patterns of vegetation productivity and biomass. We then investigate how well models driven solely by NDVI can predict the movement of the elephants, their seasonal home ranges, and the sharing of home ranges in the landscape at biweekly timescales. Finally, the implications of the results for developing conservation strategies are discussed.

## Methods

### Study area

**Figure 1:**
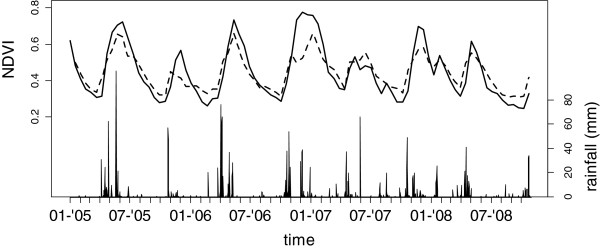
**Daily rainfall at the Marsabit meteorological station (vertical bars), observed MODIS-NDVI at 16 day intervals (solid line), and NDVI estimated from the rainfall observations using a random forest model (dashed line).**

The Marsabit protected area (2°20′N 37°20′E) comprises the Marsabit National Park and reserve. It covers 1,500 km^2^, including the dormant volcano Mt Marsabit (1680 m.a.s.l.), which is more than 1,000 m higher than its surroundings. While most of the area is covered in a mosaic of scrubland, savannah, and farmland, Mt Marsabit supports an evergreen forest covering 125 km^2^ from elevations of 1,000 m.a.s.l. and upwards. Permanent rivers are absent, but a precipitation regime of 800–1,000 mm annual rainfall, along with crater lakes, springs, and boreholes provide water in the forest year-round. In the grass and scrubland surrounding Mt Marsabit, annual rainfall is as low as 50–250 mm. Rainfall is recorded daily at a station at the edge of the evergreen forest, at 1340 m.a.s.l. (2°34′N 37°98′E) and occurs predominantly in two wet seasons: April-May (mean monthly rainfall in 2005–2008 (MMR) = 82–227 mm), and October-December (MMR = 33–109 mm, Figure 1), which are responsible for 90% of the annual rainfall. This pattern results in a longer dry season from July to September (MMR = 2–22 mm) and a shorter dry season from January to February (MMR = 1–39 mm). The national park contains evergreen forest on the mountainous slopes, which provides a dry season habitat for elephants. During the wet season, elephants occupy the scrublands at lower elevations [37].

### Elephant data

Ten elephants in the Marsabit protected area were fitted with GPS collars (manufactured by Televilt Positioning AB, Sweden). Each collar recorded the individual’s position every hour. In December 2005, 2 female and 4 male elephants that resided on the slopes of Mt Marsabit were equipped with collars. A further 2 females, and 2 males were collared in July 2006, and June 2007, respectively. Individual collars provided useful data for 207 to 648 days, with a median of 445 days. The deployment of collars was done by Kenya wildlife service (KWS), a state corporation mandated by Kenyan law (wildlife conservation and management act [amendment] of 1989) to conduct wildlife research in Kenya. GPS collar deployment does not harm the elephants and was conducted as part of the KWS management and conservation efforts of the elephant population in the Marsabit protected area.

### NDVI data

Several Earth-orbiting remote sensing instruments may be used to calculate NDVI time series. Of these, the two MODIS sensors launched in 1999 and 2002 are of particular significance to the spatiotemporal analysis of vegetation in large mammal habitats since they provide data at a 232 m resolution, and near-daily frequency free-of-charge (distributed by the Land Processes Distributed Active Archive Center, U.S. Geological Survey, Earth Resources Observation and Science Center [lpdaac.usgs.gov]). The dataset used here is compiled into 16-day cloud-free composites (MOD13, [47]). From it, changes in photosynthetically active biomass were calculated for the entire Marsabit region between 18 February 2000 and 18 February 2009. During the MOD13 production, a compositing algorithm retains from daily NDVI observations over contiguous 16 day periods, the highest-quality NDVI observation for each pixel, thereby trading data frequency for data quality [47]. Thus, within a single NDVI composite image, data recorded on any of the preceding 16 days may be represented, and intervals between consecutive observations for a single pixel may vary from 1 to 31 days. Using the exact day of acquisition for the values represented in the composite images, we converted the irregular NDVI time series to regular 16 days intervals, by first interpolating the NDVI time series for each pixel to daily resolution using univariate Akima interpolation [48] and then extracting data for every 16^th^ day.

### Describing vegetation dynamics using NDVI

Since evergreen forest only occurs on Mt Marsabit, with arid to semi-arid areas surrounding the mountain, rainfall and elevation should be important determinants of the amount of biomass and its seasonal and inter-annual change. To verify that the NDVI accurately reflects the rainfall-drive dynamics that characterizes arid ecosystem productivity, daily rainfall data collected at the meteorological station on the northwestern slope of Mt Marsabit from 2005 to 2008 were compared with the NDVI time series in the pixel coinciding with the station. A Random Forest regression model [49] was chosen to test whether NDVI could be predicted from the rainfall data, because the response of vegetation productivity to rainfall was expected to be strongly non-linear and dependent on rainfall accumulated over different time steps. Random Forest models are aggregates of regression trees, each using a random sample of the data where splits of the trees are chosen from subsets of the available predictors, randomly chosen at each node. Here, 500 trees, with at least 5 observations in each terminal node, comprised the forest built using the ‘randomForest’ package (Liaw & Cutler, 2012) in the R software (http://cran.r-project.org/). In the calibration of each tree, a third of the input data, termed the "out-of-the-bag" sample, was excluded from model calibration and used to estimate model performance.

Finally, the full MODIS NDVI record was used to describe spatiotemporal patterns in vegetation productivity in the entire Marsabit area, and in particular how the number and length of periods with high productivity (here termed ‘growth periods’), varied with elevation in the area (as extracted from a digital elevation model with a 30 m horizontal resolution derived from the Space Shuttle Radar Topography Mission (SRTM) and available through the Global Land Cover Facility (GLCF) [http://glcf.umd.edu/data/srtm/]).

### Elephants’ movements and vegetation index (NDVI)

We quantified how closely the seasonal distribution of the elephants reflects the pattern of vegetation productivity within the Marsabit area as measured by the NDVI. This was achieved by comparing the likelihood of agent-based movement models that consider different variations of the hypotheses that may describe the effects of the NDVI on elephant movement and particularly the rates at which elephants descended or ascended in the landscape. The core hypothesis, variations of which were used to generate the alternative movement models, is that the elevational migratory movements of the elephants match the rate at which vegetation productivity changes and that, in essence, the elephants are "surfing a green wave" [50, 51] by adjusting their altitude so that they remain in a narrow range of preferable NDVI. Secondly, we investigated the home range of individual elephants and the extent to which the overlap between home ranges of different elephants change during the seasonal migration cycle. In particular, we tested if the sizes of the home ranges of the elephants and the degree of overlap between the home ranges of different elephants varied as they moved between elevations.

### Individual movement modeling

For the analysis of individual movement we followed the approach developed by Bartlam-Brooks et al. [52]) that expands the approach by Bunnefeld et al. [53]. It represents alternative hypotheses regarding the drivers of animal movement through an array of competing agent-based models with increasing complexity. These models are then scored based on their ability to reproduce observed movement given known environmental conditions and without resorting to exaggerated complexity. Comparison of the qualitative scores of different models allows for the evaluation of the empirical support for the initial hypotheses. Here, this framework was used to investigate to what degree elephant movement corresponded to seasonal and sub-seasonal variations in vegetation productivity patterns, as quantified by gridded time series of NDVI. The models were parameterized and their output compared to observations of elephant’s elevation through time in order to determine whether and how elevational movement bore resemblance to spatio-temporal changes in NDVI (Table 1). In doing so, we reduced the complex 3-dimensional seasonal movement patterns to its dominant elevational characteristic [54]. The models fitted into three classes: (i) "Pure surf models" – where, following the hypothesis by Bischof et al. [51] and Fryxell and Avgar [55]), the animals move to the elevations where vegetation shows a certain level of gross primary productivity, and therefore a certain NDVI value.; (ii) "Non-linear surf model" - models that used the target NDVI to determine an elevation but further adjusted that elevation using a linear or non-linear function of the NDVI at the animal’s location, leading to an overall non-linear relationship between the target NDVI and the resulting height; and (iii) models based on (ii) but with further adjustment based on the temporal rate of change of the NDVI at the animal’s location. Two of the ten elephants showed a different movement pattern from the others; Mrs Kamau moved to the lowlands in the northwest in late October 2006 at the start of a growth period which lasted for two months. Despite a sustained period of low NDVI after this period, Mrs Kamau and Sora, a male, stayed there until mid-July 2007, when they returned to Mt Marsabit. These two individuals were excluded from the movement model analysis as they did not consistently made elevational migrations.

**Table 1:** **Alternative models, the number and values of their parameters, and the goodness of fit statistics (coefficient of determination, R** ^**2**^ **, and the Akaike information criterion, AICc) for each model**

Model type	Model	R^2^	AICc	# of par.	"Surf" _NDVI_	Additional parameters
(i)	(1) Z = {Z| NDVI = "Surf"_NDVI_}	0.056	942.7	1	0.599	
Individual	(2) Z_i_ = {Z|NDVI = ("Surf"_NDVI_)_i_}	0.245	897.2	8	0.64; 0.62; 0.46; 0.62; 0.67; 0.51; 0.63; 0.55	
Individual	(3) Z_mi_ = a × Z_i_ + b	0.501	790.7	10	Same as above	a = 0.58; b = 435.4
(ii)	(4) Z_m_ = a × Z + b	0.462	796.5	3	0.239	a = 0.66; b = 650
	(5) Z_m_ = a × (exp(b × Z))	0.46	797.6	3	0.239	a = 737.8; b = 0.00057
	(6) Z_m_ = a × Z^2^ + b × Z + c	0.462	796.6	4	0.239	a = -2.782_×_10^-5^; b = 0.70;
						c = 635.5
(iii)	(7) Z_m_ = a × (dNDVI) + b × Z + c	0.48	789.66	4	0.239	a = -0.44; b = 0.66; c = 650.6
	(8) Z_m_ = a × (dNDVI^+^) + b × (dNDVI^-^) + c × Z + d	0.49	786.46	5	0.236	a = -0.71; b = 0.086; d = 0.64;
						e = 680.4

Z is the elevational location. "Surf"_NDVI_ marks the parameterized target NDVI value the elephants will follow according to the hypothesis of "surfing the green wave". An underscore i marks an individual-based parameter set or location, Z_m_ marks the resulting modified elevational location following a linear or non-linear surf model. A vertical bar marks a condition, i.e. {Z| NDVI = "Surf"_NDVI_} means Z where NDVI equals "Surf"_NDVI_.

In all models, we determined the parameters using the function *fmincon* for non-linear constrained optimization in MATLAB version 7.9 [56]. We used randomly-chosen parameter values to initiate the optimization to avoid convergence to local minima. The Akaike Information Criterion (AICc) with a correction for sample size [57] was used to reconcile a model's likelihood by considering its coefficient of determination (R^2^) and its number of parameters and identify the most justified model. The effective sample size was reduced to the number of individuals to prevent pseudo-replication. In any case, the effectiveness of each model is only discussed relatively to the other models, which have the same sample sizes and individual numbers. All the models were globally parameterized, i.e. they did not include any individually-specific parameters. While it will be reasonable to assume that gender, rank, genetics and other individual features affect the movement behavior of each elephant, the small sample size (an unfortunate but typical characteristic of large mammal studies) did not allow for the grouping of the individuals to functional groups. Making a different model for each individual accounting for the random effects between them without any hypothetical grouping variables would deem the overall model irrelevant as its predictive power will not extend beyond the observed individuals. We nonetheless provide the parameters and statistics of such individually-based type (i) model, for reference, and to qualitatively assess the importance of the differences between individuals in determining an appropriate movement model for the population.

### Home-range analysis

Home ranges were calculated using a 95% minimum convex polygon estimator [58]. Because home range estimates can be highly sensitive to estimation methods [59], which in some cases can create spurious patterns, we also analyzed 50% minimum convex polygon estimates and 50 and 95% kernel density-based estimates which quantify the probability distribution of animal's use of space [60, 61]. All home range estimates were made using the *adehabitat*[62] library for R. For the calculation of the NDVI in the elephants’ habitat, we outlined for each animal their year-round home range (including both rainy and seasons). To analyze the change in home range sizes and overlaps we calculated monthly home range sizes if at least 75% of the potential hourly geo-location recordings were available during an interval. To estimate how much of their home range animals shared with other elephants, we relied on the utilization distribution overlap index (UDOI, [60]). Based on the Hurlbert index [63], it measures the amount of overlap in utilization distributions, relative to two animals using the same space uniformly. Values below 1 indicate that the observed overlap is smaller compared with uniform space use, whereas values above 1 indicate higher than normal overlap relative to uniform space use. The overlap of home ranges was only calculated in months when a home range estimate was available for at least three animals. Correlations between the degree of overlap between home ranges and spatial or temporal variables were quantified using Kendall’s τ, a non-parametric measure of association which is rank-based [64] and therefore insensitive to bias in home range overlap estimates.

## Results

### Rainfall-vegetation dynamics inferred from NDVI

At 32 day intervals, NDVI increases with rainfall accumulated in the previous month (r = 0.53, p < 0.01, n = 43), but the NDVI reflects the ability of plants and soils to store water and thus responds to short as well longer term rainfall patterns: a Random Forest model accurately predicted NDVI values at 16 day intervals (n = 86), based on the NDVI value 16 days earlier and the rainfall accumulated in the past week and month (the model explained 84% of the variance in NDVI based on the "out-of-the-bag" samples, Figure 1). The start and end of growth periods are characterized by marked changes in NDVI [41]. When considering only 16 day intervals where the NDVI had decreased or increased by at least 0.05, i.e., changes between wet and dry periods, the model explained respectively 78% (n = 28) and 60% (n = 21) of the variance in NDVI.

### Vegetation dynamics in the Marsabit area

Higher elevations in the Marsabit area experience more days with vegetation growth1

where G is a proxy of the period with green vegetation and is defined as the number of days with NDVI > 0.45 per year, and ALT indicates the elevation in km (r^2^ = 0.63, RMSE = 33 days, n = 2500).

**Figure 2:**
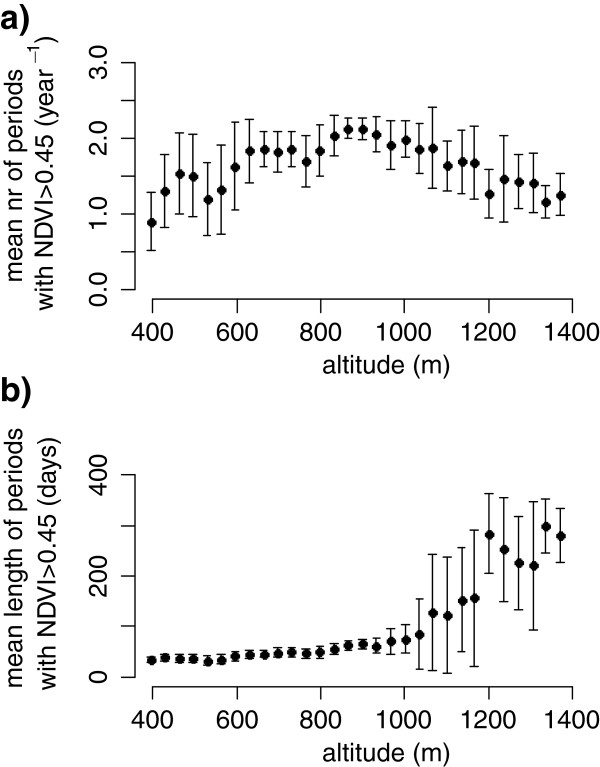
**Descriptive statistics of the annual growing periods in the Marsabit area as a function of elevation. a)** The number of growth periods per year, estimated as NDVI > 0.45, and **b)** the mean length of these seasons, and associated standard deviations.

This is the result of variability in the number of growth periods each year as well as their length (Figure 2). The lower elevations of the Marsabit area (< 650 m.a.s.l.) experience one very short growth period per year, generally lasting less than a month, or go through years without any growth periods at all. Elevations of 650 to 1,100 m.a.s.l., generally experience two growth periods per year. Above 1,100 m.a.s.l., the NDVI reflects the presence of evergreen trees with NDVI values dropping below 0.45 only once per year or two years (Figure 2a), and for only short periods of time. Consequently, NDVI-derived growth periods can last for a year or more at the higher elevations on Mt Marsabit (Figure 2b).

### Elephants’ migrations and the flushing and senescing of vegetation

**Figure 3:**
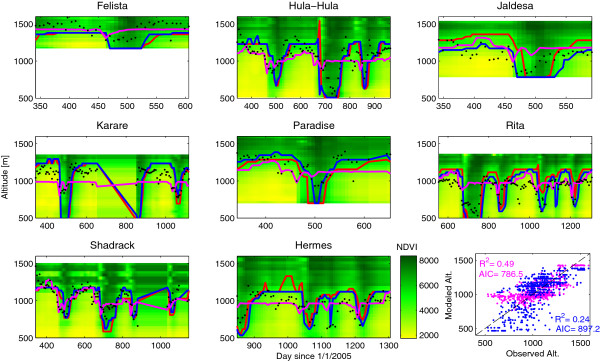
**NDVI and elevational (y-axis, meters) migration of the ten elephants equipped with GPS-collars in the Marsabit area.** Color map in the background shows NDVI as a function of height and time (x-axis, in days since the start of the experiment). Dots show the observed elevations of the elephants and the bold lines show the predictions of 3 models. Red line marks the pure surf model (Table 1 model 1), black line marks the individual surf model (Table 1 model 2) and cyan line marks the most justified model we found, which include linear modification of a single "Surf"-NDVI with linear effects of positive and negative temporal changes to NDVI (Table 1, Model 8). Each modeled individual is shown is a different panel. The bottom right panel shows the overall goodness of fit of two of the models, model 8 in cyan and model 2 in black.

The elevational migration of individual elephants very closely matches the spatiotemporal patterns in flushing and senescing of vegetation in their year-round home range (Figure 3), although not all the animals lived in the same elevational or NDVI range. In general, the elephants tracked an intermediate value of NDVI. Table 1 shows the models and parameters that describe this movement. Elephants adjusted their elevation upward during the dry seasons when no green vegetation was available in the lower regions of their home range. As soon as vegetation flushed at the lower elevations, the elephants rapidly descended from the Marsabit forest (Figure 3). Depending on the abundance of fresh biomass (as approximated through NDVI) at lower elevations, the animals migrated further down the mountain. As long as productive vegetation was available at the lower elevations, the animals generally did not return to the higher elevations of their home range. Instead, the timing of their return towards the evergreen forest matched the senescence of the vegetation, which occurred first at lower elevations. The elephants, however, did not move towards locations with a peak NDVI, typically higher in the mountains than their recorded position. Instead they overlapped an intermediate range of NDVI (0.59, Table 1, model 1) which approximately corresponds with the elevation where the spatial vertical gradient of NDVI is maximal (Figure 3). This behavior corresponds with "surfing the green wave" hypothesis [50, 51]. The simplest "pure-surf" model assumes that there is a fixed NDVI where the elephants will be located. We constructed the model by programming a moving "agent" that scans the virtual landscape of the NDVI field vertically and climbs to the height were this value first occurs. We optimized the model to find an NDVI value that will drive a model with the highest overall fit to the observation we have of the entire population's location. This model (Table 1, Model 1; and red line in Figure 3) has a very low coefficient of determination (0.056). The reason is that some elephants, such as Felista, spend most of their time at relatively higher elevations with higher mean NDVI while other prefer to stay at lower elevations and lower NDVI values (e.g., Jaldesa, Hermes). A straight forward single NDVI value with which the elephants "surf" the environment is, thus, not a good descriptor of their movement. Hypothetically, this could be a result of the differences between the elephants.

An individual-based surf model that finds a different surf-NDVI for each elephant has an R^2^ = 0.24 (Table 1, Model 2; cyan line in Figure 3). Elephants ranged quite widely in their apparent NDVI preference (between 0.46 and 0.67, which encompasses roughly 30% of the observed range of NDVI for that area). However, such a model has 8 parameters. A much more effective model includes a linear scaling of the elevation that is predicted by a single over-all-individuals NDVI, and achieves a higher R^2^ =0.46 and lower AICc with only 3 parameters (Table 1 Model 4). This indicates that there are strong common characteristics across the population to the way the elephant movement relates to NDVI that explain the movement better that assuming each individual follows different rules. The meaning of such linear scaling of the "surf" model is that the elephants choose a lower target NDVI to "surf" (0.23 in this model) but overshoot the location where that value is found by a fixed elevation (650 m) and then further adjust their location based on the elevation, with a decreased tendency of going further up the higher they are (indicated by the model liner-slope coefficient = 0.66 that is smaller than 1). We tested other modification models (exponential, polynomial) but they were less justified (higher AICc) than the linear model (Table 1, Models 5–6).

We tested the effect of the time-rate of change of NDVI (dNDVI). Including a modification for the elevational location based on dNDVI further improved the models, and the AICc indicates that this improvement was justified, indicating that the rate of change provides additional queues to the elephant's movement behaviour (Table 1, Models 7–8). Including a linear effect of dNDVI significantly improved the model. The best model was achieved when positive and negative dNDVI were included separately with independent linear effects (Table 1 Model 8). This model (R^2^ = 0.49, AICc = 786.46) showed that the elephants prefer going downward when there is a fast temporal change of NDVI, farther than the elevation that would have been predicted by the contemporary NDVI value. Similarly they prefer to climb when dNDVI is negative and indicates a dry spell or the end of the green season. Surprisingly, we found that this model is very effective at predicting the mean height at which each elephant will be present but underestimates the movement of the elephants around this mean elevation, while simpler "surf" models overestimate the variation in the elephant's movement (Figure 3). It is interesting to note that this model is more justified (with lower AICc, albeit slightly lower R^2^) than a linearly-modified individual surf model (Table 1 model 3), providing further indication to the common characteristics of movement with respect to NDVI.

### Sizes and overlaps of home ranges

Similar to the movement models, the analyses of home range sizes and overlap were restricted to the seven elephants that returned to the Marsabit forest every dry season, excluding the two animals that made the longer migration to the northwest of the area. When at higher elevations, elephants had smaller home ranges:2

where HR is the monthly home range size (in km^2^) of an animal, and ALT the mean elevation of the animal during that period (n = 80).

Similar regression analyses, considering the home ranges of individual animals, showed a statistically significant decrease in monthly home range size with elevation for five of the seven elephants (Parameter Estimate (PE) = -0.0005 ± 0.00020 to -0.0065 ± 0.0022). For the other two animals (Paradise and Rita) the regression suggested a small increase in monthly home range size with elevation (PE = 0.00020 ± 0.00027 and PE = 0.00091 ± 0.00085). Calculating home ranges as 50% convex hull polygons, or 50% or 95% of the kernel-estimated utilization distributions, rather than as 95% convex hull polygons, didn't alter home range-altitude relationships observed across animals (-0.0025 < PE < -0.0017, SE < 0.0007). The home range change individual animals display in response to elevation was also robust across estimation methods with all but the same two animals showing significant decreases in home range at higher altitudes irrespective of estimation method.

Only two data samples were available to assess the overlap between monthly home ranges of different elephants: sample 1 comprised data for Paradise, Karare, Jaldesa, Hula_Hula, Felista and spanned 5 months between January and August 2006, and sample 2 comprised data for Rita, Karare, and Hermes spanning June, August, and December of 2007. Overlaps between home ranges increased consistently with home range sizes in both samples (τ = 1), irrespective of home range estimation method, and appeared to be smaller at higher elevations (sample 1: τ = -0.8, sample 2: τ = -0.3).

## Discussion

### Spatio-temporal patterns of vegetation productivity and elephant movement

Rainfall and other sources of water are the major determinant of variation in primary production, and thus the availability of forage to herbivores, within and across African ecosystems [65]. The Marsabit area reflects this variation spatially as well as seasonally: owing to the continuous availability of water on Mount Marsabit, it supports an evergreen forest, while lower elevations experience progressively fewer and shorter growth periods, with the most arid areas showing years without significant vegetation growth. Our analyses reveal how strongly the movements of elephants in the Marsabit area are linked to these spatio-temporal patterns in vegetation productivity.

**Figure 4:**
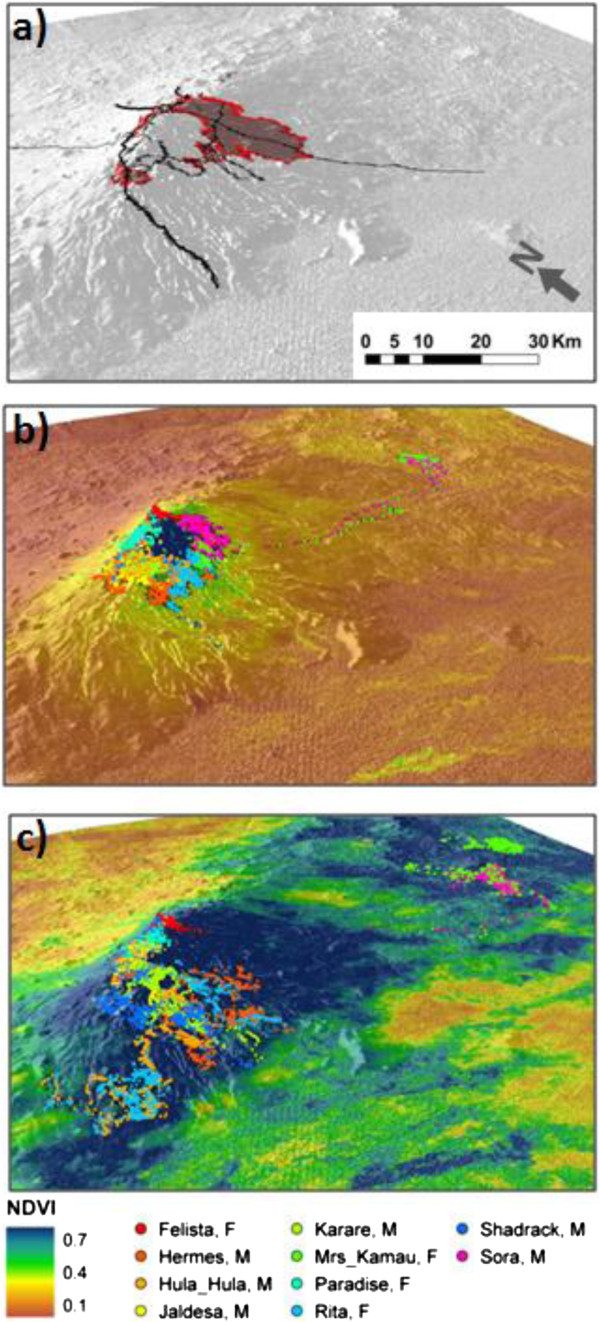
**The Marsabit study area and recorded elephant movements. (a)** with agricultural areas (shaded and outlined in red), major roads (bold lines), minor roads (thin lines), and elephant locations recorded in **(b)** December 2005–2008 on a background of NDVI measured between December 19 and 31 December 2006, and **(c)** July 2006–2008 on a background of NDVI measured between 12 and 28 July 2006, draped over a digital elevation model.

The movement of elephants tracked the productivity response of vegetation following not only the large rainfall events, but also of the smaller ones, with timing, duration and speed matching the greening and senescing of the vegetation. The elephants very rarely ascended Marsabit Mountain while green vegetation was available at lower elevations. Occasionally, animals descended to the lowlands when no increase in vegetation productivity was indicated by the satellite data, which might be due to cloud cover preventing the detection of short-lived greening of vegetation. The general migration pattern was not followed by two of the ten elephants, namely Mrs Kamau and Sora. They spent long periods of the drier season at lower elevations in the northwest of the Marsabit area, over 90 km from the Marsabit forest. This area is characterized by lava rock outcrops and pan shaped shallow depressions that capture water during the rainy season and hold it long into the dry season if rainfall is more sustained than normal, as was the case in December 2006 (Figure 4).

### Contrasting landscape use in dry and wet seasons

While the Marsabit forest provides water and a green canopy year-round, as indicated by consistently high NDVI values, trees are tall (>20 m) and undergrowth and shrubs are sparse. Consequently, forage is unavailable to the elephants or of poor quality, compared to the grasslands and scrublands at lower elevations [37]. The immediate link between landscape phenology and rainfall in combination with the observed migration pattern suggests an opportunistic migration strategy where the elephants reside in the savannah and scrublands as long as the availability of forage and especially water allows, and move to the forest when it does not or occasionally stay close to other sources of water. This strategy agrees with evidence that the dry season to wet season transition concurs with a habitat range expansion away from year-round water sources in Botswana [30], as well as northern Kenya where it further coincides with a greater dominance of grasses in the elephant diet during times of peak NDVI [33]. Holdo et al. [66] hypothesized that a negative correlation between water availability in the landscape and the peak nutritional value of vegetation drive many of the long-distance ungulate migrations observed in African savanna landscapes, based on a study of Wildebeest migration. A similar acceleration at the end of the dry season has been reported by Owen-Smith and Ogutu [67] and Birkett et al. [24] signifying a release from the “dry season bottleneck” [68]. While we didn't assess nutrient levels in vegetation directly, the grasses and shrubs that dominate vegetation composition at the lower elevations of the Marsabit area should provide better forage than the forest during the rainy season. Our results thus lend support to the aforementioned hypothesis, extending it to non-ungulates and migratory behavior over much smaller distances; the two main corridors between wet and dry season elephant habitats in the Marsabit area are about 20 and over 90 km long [37].

The Marsabit forest measures only 125 km^2^, which is less than 10% of the Marsabit protected area. Yet all ten elephants we followed resided in the Marsabit forest during two or more dry seasons. During these periods, the individual animals ranged over much smaller areas each month, than during wet seasons (monthly home range: 0.68 ± 0.12 km^2^ vs. 1.18 ± 0.15 km^2^). This result confirms the previously observed differences in the size of the combined dry season and wet season ranges (907 km2) of all tracked elephants in the Marsabit area (266 km2 vs 907 km2; [37]) and matches a pattern observed in other elephant populations [25, 28, 30]. While these results are to be expected given the limited size of the Marsabit mountain top, population-level generalizations about the spatio-temporal changes in home range sizes, including gender-differences, and what drives them, is beyond the scope of this manuscript.

Limited water availability seasonally confines the elephants within Marsabit forest. Accordingly, when rain accumulates and causes vegetation to flush at lower elevations, elephants are quick to descend from the forest; as observed by Ngene et al. [37] individual elephants in the Marsabit area move at speeds below 0.2-1 kmh^-1^ within their seasonal habitats, but travel faster than 1 kmh^-1^ when moving between them [37]. As they reach the lower elevations, they occupy larger areas (450–470 km^2^; [37]). When moving back to their dry season range around the mountain, they encounter farms located at the higher elevations. This return movement coincides with maturing of crops, grown in farms along the elephant’s range, resulting in crop damage by the elephants [37, 69].

## Conclusions

### Management implications

Surface-water availability has the potential to limit the size of elephant populations, as was suggested for the Hwange National Park in Zimbabwe [70]. In the Marsabit area, apart from isolated water holes, only the Marsabit forest appears to provide a sufficient and reliable supply of water during the dry season. The establishment of an ecological relevant number of water holes in the Marsabit area therefore could reduce the reliance on the forest in the dry season and ultimately benefit the elephant population. However, placement of such water holes needs to be carefully considered as similar manipulations have led to habitat loss in the vicinity of water holes [71]. Furthermore, they could increase human-animal interactions as the animals currently migrate to and from the lowland scrublands at the start and end of each wet season along corridors amid settlements and farms that border Marsabit forest [36, 37]. Any manipulations to provide additional dry season habitat, thus needs to be traded off with the potential for increased exposure to predators, illegal hunting or habitat degradation [72]. Our results indicate that the limited size of the forest and its poor quality of forage compared to the scrublands currently affects the elephants’ ranging behavior through competition. Any further encroachment on the Marsabit forest and its surroundings by agricultural activity or environmental change is therefore likely to increase the stress on its elephant population and human-wildlife conflict (see for example, [73]). Finally, the highly opportunistic migration pattern with regard to rain-driven vegetation dynamics displayed by the Marsabit elephants, indicates that any changes in the precipitation regime will immediately be reflected in the elephants’ habitat use.

Environmental change, man-made land cover alteration [29, 74, 75], and exceptional events, such as fire and el Niño [76], can cause dramatic changes in the way animals utilize the landscape. A combination of GPS tracking data and NDVI-derived estimates of productivity, can produce base-lines or help detect changes in this utilization function in near real-time. and can now be achieved using an automated interface through the Environmental-Data Automated Track Annotation (Env-DATA) system in MoveBank (http://www.movebank.org) [77] .More generally, it can increase our understanding of the migration of most species in seasonal ecosystems [78]. The information gained on their interaction with landscape-scale biophysical dynamics in particular can prove of great value for management and conservation strategies.
